# Linking phytoplankton community metabolism to the individual size distribution

**DOI:** 10.1111/ele.13082

**Published:** 2018-05-25

**Authors:** Daniel Padfield, Angus Buckling, Ruth Warfield, Chris Lowe, Gabriel Yvon‐Durocher

**Affiliations:** ^1^ Environment and Sustainability Institute University of Exeter Penryn Cornwall TR10 9EZ UK; ^2^ Centre for Ecology and Conservation College of Life and Environmental Sciences University of Exeter Penryn Cornwall TR10 9FE UK

## Abstract

Quantifying variation in ecosystem metabolism is critical to predicting the impacts of environmental change on the carbon cycle. We used a metabolic scaling framework to investigate how body size and temperature influence phytoplankton community metabolism. We tested this framework using phytoplankton sampled from an outdoor mesocosm experiment, where communities had been either experimentally warmed (+ 4 °C) for 10 years or left at ambient temperature. Warmed and ambient phytoplankton communities differed substantially in their taxonomic composition and size structure. Despite this, the response of primary production and community respiration to long‐ and short‐term warming could be estimated using a model that accounted for the size‐ and temperature dependence of individual metabolism, and the community abundance‐body size distribution. This work demonstrates that the key metabolic fluxes that determine the carbon balance of planktonic ecosystems can be approximated using metabolic scaling theory, with knowledge of the individual size distribution and environmental temperature.

## Introduction

Phytoplankton is responsible for around half of the carbon fixed by the biosphere, despite accounting for < 1% of global autotrophic biomass (Falkowski 1994; Field 1998). Most of this carbon, fixed through photosynthesis, is quickly remineralised by respiration (Falkowski *et al*. 2000) and the difference between community respiration (CR) and gross primary production (GPP) represents the amount of carbon that an ecosystem can sequester from the atmosphere (Falkowski *et al*. 2008). Despite its importance to the global carbon cycle, methods of measuring planktonic metabolism *in situ* (through monitoring gas concentrations) and *in vitro* (e.g. traditional bottle‐incubation measurements) have led to contrasting conclusions as to whether CR generally exceeds GPP in freshwater (del Giorgio *et al*. 1997; Carignan *et al*. 2000; Duarte & Prairie 2005) and marine ecosystems (Duarte *et al*. 2011; Williams *et al*. 2013). If CR > GPP, aquatic ecosystems are net heterotrophic and release more CO_2_ to the atmosphere through respiration than they fix via photosynthesis (del Giorgio & Peters 1994; Duarte & Prairie 2005; Duarte *et al*. 2011).

With the aim of better understanding the key fluxes of carbon in aquatic communities, previous work has attempted to link individual physiology and community size structure (del Giorgio & Gasol 1995; del Giorgio *et al*. 1999) to predict phytoplankton community metabolism (López‐Urrutia *et al*. 2006; Yvon‐Durocher & Allen 2012; Zwart *et al*. 2015). One successful approach has been the application of metabolic scaling theory (MST), which links organism and ecosystem metabolism using the relationships between temperature, body size and metabolic rate (Enquist *et al*. 2003; Brown *et al*. 2004; Allen *et al*. 2005). MST has previously helped explain and predict the impact of warming on population, community and ecosystem‐level phenomena (Enquist *et al*. 2003; Savage *et al*. 2004; O'Connor *et al*. 2009; Pawar *et al*. 2012).

At the community‐level, GPP tends to be less sensitive to changes in temperature than CR (López‐Urrutia *et al*. 2006; Anderson‐Teixeira *et al*. 2011; Regaudie‐De‐Gioux & Duarte 2012; Yvon‐Durocher & Allen 2012). Consequently, MST has been used to predict that increasing temperatures will shift the metabolic balance of phytoplankton communities towards heterotrophy (López‐Urrutia *et al*. 2006; Regaudie‐De‐Gioux & Duarte 2012), which could act as a positive feedback with climate warming. However, this work only considered the direct effects of warming on rates of photosynthesis and respiration – for example, via increases in the amount of kinetic energy available for driving chemical reactions. Temperature can also indirectly influence phytoplankton community metabolism through structural changes to the community such as shifts in biomass (Chust *et al*. 2014; Yvon‐Durocher *et al*. 2015), biodiversity (Hillebrand *et al*. 2012; Lewandowska *et al*. 2012, 2014; Yvon‐Durocher *et al*. 2015), community composition (Markensten *et al*. 2010; Thomas *et al*. 2012) and size structure (Daufresne *et al*. 2009; Morán *et al*. 2010; Yvon‐Durocher *et al*. 2011). In addition, local adaptation of organisms to environmental temperature can alter metabolic rates (Berry & Bjorkman 1980). These indirect impacts of warming can be as large as the direct effects of temperature on community metabolism (Padfield *et al*. 2017). To improve predictions of the impacts of warming on phytoplankton community metabolism, it is essential to consider these indirect effects of warming alongside the direct effect of temperature on metabolic rates.

Previous studies linking temperature, community structure and metabolism in phytoplankton have focused on a subset of these direct and indirect relationships in isolation. For example, previous work has explored the relationship between body size, metabolic rate and abundance (Huete‐Ortega *et al*. 2012; Huete‐Ortega *et al*. 2014; García *et al*. 2016); body size and temperature (Morán *et al*. 2010; López‐Urrutia & Morán 2015); and metabolic rate, temperature and biodiversity (Lewandowska *et al*. 2012; Yvon‐Durocher *et al*. 2015). How community‐level metabolism emerges from the direct effect of temperature on individual physiology, and the indirect effects of temperature on community structure and body size remains largely unexplored. Here, we investigate how GPP and CR in phytoplankton communities are influenced by the direct effect of temperature on individual photosynthesis and respiration and the indirect effect of temperature on community structure (i.e. size structure, composition and abundance). We do this by testing the predictions of a model derived from metabolic scaling theory against empirical data from a warming experiment with phytoplankton communities.

## Theory

Metabolism sets the pace of the life (Brown *et al*. 2004) and is a key process that can link patterns and processes across levels of organisation by quantifying the relationships between metabolic rate, body size and temperature (Gillooly *et al*. 2001; Enquist *et al*. 2003; Brown *et al*. 2004). The central equation from metabolic scaling theory (MST) predicts individual metabolic rate (i.e. photosynthesis or respiration), *b*_*i*_, at environmental temperature, *T* (in Kelvin).

(1) $$b_{i}\left(T\right)=b_{i}\left(T_{c}\right)m_{i}^{\alpha }e^{E\left(\frac{1}{kT_{c}}\;-\;\frac{1}{\mathrm{kT}}\right)}$$

This modified Boltzmann formulation is only valid below the optimum temperature of the organism, and thus assumes that taxa predominantly experience temperatures lower than their optimum temperature. The temperature centring of the data $\left(\frac{1}{kT_{c}}-\;\frac{1}{\mathrm{kT}}\right)$, where *T*_*c*_ is a common temperature (in K) and *k* is Boltzmann's constant (8.62 × 10^−5^ eV K^−1^) sets the normalisation constant, $b_{i}\left(T_{c}\right),$ at a biologically relevant temperature instead of 0 K (−273.15 °C) (Yvon‐Durocher & Allen 2012; Padfield *et al*. 2016). $m_{i}^{\alpha }$ is the mass dependence of metabolic rate characterised by an exponent, *α*, which is thought to reflect mass‐dependent changes in the density of metabolic organelles (Allen *et al*. 2005). The exponent, *α*, was originally considered to be three quarters across all organisms (Gillooly *et al*. 2001; West *et al*. 2002), but recent empirical studies have found the size‐scaling exponent to be steeper and close to isometric (*α* = 1) in phytoplankton (Marañón 2008; Huete‐Ortega *et al*. 2012) and super‐linear in bacteria (*α* > 1) (DeLong *et al*. 2010; García *et al*. 2016). *E* (eV) is the activation energy that describes the temperature dependence of metabolism. Previous work indicates that the activation energy of gross photosynthesis tends to be weaker than that of respiration across both terrestrial and aquatic autotrophs (Allen *et al*. 2005; Anderson‐Teixeira *et al*. 2011; Padfield *et al*. 2016; Schaum *et al*. 2017).

The effects of body size and temperature on individual metabolic rate in eqn (1) are summed across all individuals within a community, *j*, to yield an estimate of total community metabolic rate (Enquist *et al*. 2003; Allen *et al*. 2005; Yvon‐Durocher & Allen 2012):

(2) $$B_{j}\left(T\right)=B_{j}\left(T_{c}\right)e^{E\left(\frac{1}{kT_{c}}\;-\;\frac{1}{\mathrm{kT}}\right)}$$

where $B_{j}\left(T\right)$ is the rate of metabolism of community *j*, at temperature *T*, in Kelvin (K). $B_{j}\left(T_{c}\right)$is the community‐level normalisation ($=\sum_{i=1}^{n_{\mathrm{tot}}}b_{i}\left(T_{c}\right)m_{i}^{\alpha })$ at *T*_*c*_ (= 18 °C [291.15 K]), where *n*_*tot*_ is the total number of individual organisms, *i*, that comprise all the organisms in *j* and accounts for the abundance, size structure and the average individual‐level metabolic normalisation constant.

Total biomass, $M_{\mathrm{tot}}=\;\sum_{i=1}^{n_{\mathrm{tot}}}m_{i}$, has been used to predict community respiration (Robinson *et al*. 2002a,b) and gross primary production (del Giorgio *et al*. 1999). However, eqn (2) demonstrates that if the size‐scaling of metabolism is not isometric (α≠1), then total biomass and community metabolism will not be directly proportional (Allen & Gillooly 2009). By multiplying total biomass, *M*_*tot*_, with a biomass‐weighted average of the relationship between body size and metabolic rate, $m_{i}^{\alpha -1}\left(=\left(\sum_{i=1}^{n_{\mathrm{tot}}}b_{i}\left(T_{c}\right)m_{i}^{\alpha }\right)/\left(\sum_{i=1}^{n_{\mathrm{tot}}}m_{i}\right)\right)$, mass‐corrected biomass, $M_{\mathrm{tot}}\left(m_{i}^{\alpha -1}\right)$, can account for the size‐scaling of metabolic rate with body size. Mass‐corrected biomass is predicted to be proportional to the total metabolic capacity (i.e. GPP or CR) of the biomass pool of a community (Allen *et al*. 2005; Yvon‐Durocher & Allen 2012; Yvon‐Durocher *et al*. 2012). By rearranging eqn (2) to control for the direct effect of temperature, *T*, on metabolism, mass‐corrected biomass can be used to compare metabolism estimates among communities that differ in size structure, standing biomass and environmental temperature (Barneche *et al*. 2014).

(3) $$\frac{B_{j}\left(T\right)}{e^{E\left(\frac{1}{kT_{c}}\;-\;\frac{1}{\mathrm{kT}}\right)}}=M_{\mathrm{tot}}\left(m_{i}^{\alpha -1}\right)$$

In eqn (1), $M_{\mathrm{tot}}\left(m_{i}^{a-1}\right)$ is an estimate of the metabolic flux of the community that incorporates any effect of temperature on the individual metabolic normalisation constant. This is necessary because terrestrial and aquatic autotrophs can up‐regulate their metabolic normalisation constants at low temperature, and down‐regulate them at high temperature to compensate for the constraints of thermodynamics on enzyme kinetics (Atkin *et al*. 2015; Padfield *et al*. 2016, 2017; Reich *et al*. 2016; Scafaro *et al*. 2017). After controlling for the direct effect of temperature, *T*, eqn (1) predicts that temperature‐corrected GPP and CR should be directly proportional (the slope of the log‐log relationship should be 1) to the mass‐corrected biomass of the community. However, this key prediction of MST is difficult to test experimentally as measuring community metabolism and the complete size distribution of all organisms in an ecosystem simultaneously is logistically challenging (Yvon‐Durocher & Allen 2012).

In the MST framework described in eqn (2), variation in total abundance, *n*_*tot*_, between communities will also alter total metabolic rates. MST predicts that under steady state conditions, carrying capacity should be inversely related to mass and temperature (Savage *et al*. 2004); changes in $n_{\mathrm{tot}}$ are expected to be intrinsically linked to the effects of body size and temperature on metabolic rate (White *et al*. 2007). Under constant resource conditions, eqn (2) predicts a trade‐off between shifts in community size structure, temperature and total community abundance (Enquist *et al*. 2003). These trade‐offs within and across communities are similar to Damuth's rule and ideas of energetic equivalence, where the scaling of abundance and body size is the inverse of the size‐scaling of metabolic rate (Damuth 1981; White *et al*. 2007).

At steady state, any increase in the average metabolic rate of the individuals within a community (e.g. via rising temperature or shifts in size structure) should result in a proportional decrease in the total number of individuals (the slope of the log‐log relationship should be −1) (Enquist *et al*. 1998; White *et al*. 2004; Ernest *et al*. 2009). We refer to this trade‐off between the number of organisms and the average individual‐level metabolic rate as ‘community‐level metabolic compensation’. This compensation means that total community metabolism can remain unchanged in the face of shifts in environmental temperature owing to adjustments in the abundance, biomass and size structure of communities.

We now test this framework and its predictions using measurements of GPP and CR of phytoplankton communities from a mesocosm experiment in which communities had been either experimentally warmed (+ 4 °C) or left at ambient temperature for over 10 years. Specifically, we test whether community metabolic rate can be estimated from the individual size distribution and the predictable relationships between body size, temperature and individual physiology (see eqn (2)). We then examine whether changes in abundance, biomass and size structure compensate for the direct effects of temperature on individual metabolic rates, buffering the response of total community metabolism to warming. Critically, by inoculating microcosms from a long‐term mesocosm experiment using a fully factorial design and measuring each microcosm's metabolic flux and individual size distribution, we can investigate both the direct and indirect effects of warming on phytoplankton community metabolism.

## Methods

### Experimental setup and maintenance

Twenty freshwater mesocosms, each holding 1 m^3^, were set up in 2005 to mimic shallow lake ecosystems (Yvon‐Durocher *et al*. 2010). They are situated at the Freshwater Biological Association's river laboratory (2° 10′ W, 50° 13′ N) in East Stoke, Dorset, UK. Of the 20, 10 mesocosms have been warmed by 4 °C above ambient temperature for more than 10 years. We sampled all 20 mesocosms (*c*. 200 mL) and inoculated each sample into laboratory microcosms in a reciprocal transplant experiment on 13 April 2016. Microcosms (200 mL) were inoculated with a starting density of 200 cells mL^−1^ and placed in incubators (Infors‐HT) at 16 and 20 °C (the temperatures of the ambient and warmed mesocosms, respectively, on the day of sample collection). This resulted in 40 communities with 10 replicates of each combination of short‐ and long‐term warming (i.e. warmed mesocosm in warm incubator, ambient mesocosm in ambient incubator, warmed mesocosm in ambient incubator and ambient mesocosm in warm incubator) (see supplementary methods for a more detailed description of the mesocosms and experimental setup).

### Quantifying community diversity

We quantified microbial community composition and diversity by sequencing the V4 hyper‐variable region of the 16S rRNA gene at the end of the experiment. On the day of metabolic rate measurements, 50 mL of each community was centrifuged at 2000 g for 45 min at 4 °C. The supernatant was then removed, and the pellet transferred to 1.5 mL Ependorf tubes and centrifuged again for 30 min at 15000 g. The supernatant was again removed, and the samples were frozen at −80 °C prior to DNA extraction. DNA was extracted from samples using a Qiagen DNeasy Plant Mini Kit (Qiagen, Düsseldorf, Germany) following the manufacturer's instructions. Genomic DNA was further purified and concentrated using Agencourt AMPure XP beads (Beckman Coulter, CA, USA) at a ratio of 1:1.4. The products of this clean up were eluted into *c*. 25 μL 10 mM TRIS. Subsequent polymerase chain reaction (PCR) amplification and sequencing of the 16S V4 region was undertaken by the Centre for Genomic Research (Liverpool, UK) following the Illumina MiSeq 16S Ribosomal RNA Gene Amplicons workflow.

Sequence data was analysed in R (v 3.3.2) (Team 2014) using the packages *‘dada2’* and ‘*phyloseq’* (Callahan *et al*. 2015, 2016), following the full stack workflow to estimate error rates, infer and merge sequences, construct a sequence table, remove chimeric sequences and assign taxonomy (Callahan *et al*. 2016). We assigned autotroph taxonomy using PhytoREF (Decelle *et al*. 2015): a reference database for the 16S rRNA gene contained in the plastid of photosynthetic eukaryotes (see supplementary methods for a full description of the sequencing data analysis protocols). Samples were removed if represented by fewer than 1000 reads and the remaining samples were standardised to the total number of amplicon sequence variants [ASVs; (Callahan *et al*. 2017)] through rarefaction to account for biases associated with differences in sequencing effort. We then selected ASVs corresponding to autotrophic taxa, which resulted in samples from 37 of the 40 communities that could be used for downstream analysis.

### Measuring community metabolism and community size structure

After *c*. 30 days of culture, we measured community metabolism at incubator temperature (16 or 20 °C). Aliquots (30 mL) of each community were concentrated through centrifugation (*c*. 500 g for 30 min at 4 °C), re‐suspended in 5 mL of fresh culture medium, and acclimatised to the measurement temperature for 15 min in the dark prior to measuring metabolic flux. Primary production was measured as oxygen evolution at multiple light intensities to characterise a photosynthesis‐irradiance (PI) curve for each community (Fig. S1). Community respiration was measured as oxygen consumption in the dark at the end of each PI curve to ensure respiration was not limited by available photosynthate during the measurement period. Each individual PI curve was fit to a modification of the Eiler's curve for photoinhibition (Eilers & Peeters 1988) (see supplementary methods).

The community size distribution was measured by flow cytometry using the sample from the respirometer immediately after metabolic rate measurements. Cell size was calculated by converting values of forward scatter from the flow cytometer into values of diameter (*d*; μm) (Schaum *et al*. 2017). The biovolume of each cell was then calculated by assuming each particle was spherical $\left(\text{biovolume}=\;\frac{4}{3}\;\pi \;{\left(\frac{d}{2}\right)}^{3}\right)$ and converted into units of carbon (μg cell^−1^) using a conversion factor of 0.109 × 10^−6^ (Montagnes *et al*. 1994). We also quantified heterotrophic bacterial abundance using a SYBR gold stain. Bacteria represented < 5% of total carbon biomass in all but one of the microcosms (Fig. S2) and are therefore unlikely to have significantly impacted the measurements of community metabolism. Thus, all analyses used only the size distribution of the autotrophic communities.

### Statistical analyses

To compare the composition of phytoplankton communities, we examined the impact of short‐ and long‐term warming on the Bray–Curtis distance, which compares the compositional dissimilarity between samples based on abundances. Differences in composition between communities were explored using the rarefied samples of each community and Bray–Curtis distance using the R packages ‘*phyloseq’* (Callahan *et al*. 2016) and ‘*vegan’* (Oksanen *et al*. 2018). Permutational anova tests were run using the ‘*adonis*’ function from the ‘*vegan’* package in R using short‐ and long‐term warming as main effects and Bray–Curtis distance as a response term with 9999 permutations.

To estimate primary production, we fit eqn S1 to the measurements of oxygen flux using nonlinear least squares regression using the *R* package ‘*nls.multstart*’ (Padfield & Matheson 2018). This method of model fitting involved running up to 1000 iterations of the fitting process with start parameters drawn from a uniform distribution and retaining the fit with the lowest Akaike Information Criterion (AIC) score. Estimated gross primary production at light saturation (see supplementary methods) and measured community respiration were used in the metabolic scaling framework. We analysed the effect of short‐ (ambient or warm incubator) and long‐term warming (ambient or warm mesocosm) on total community metabolism using an Analysis of Covariance in a mixed effects model framework. A random effect was included to account for the hierarchical structure of the data (laboratory microcosms nested within long‐term mesocosms). Separate models were conducted for each metabolic flux and model selection was carried out by comparing nested models using likelihood ratio tests.

We assessed the ability of our model (eqn (2)) to estimate community metabolism from the direct effects of warming on individual metabolic rate and changes in the individual size distribution using a maximum likelihood (ML) approach in two stages. We first fitted eqn (2) to the measurements of GPP, CR, the individual size distribution of each community ($\sum_{i=1}^{n_{\mathrm{tot}}}b_{i}\left(T_{c}\right)m_{i}^{\alpha })$) and the incubator temperature (expressed as $\left(\frac{1}{kT_{c}}-\;\frac{1}{\mathrm{kT}}\right)$, where *T*_*c*_ = 18 °C). We treat the key parameters in eqn (2) (e.g. the metabolic normalisation constant, size‐scaling exponent and activation energy) as unknown and use the ML approach to simultaneously estimate the values that yield the best fit (maximise the likelihood function) of the model, given the data. Because the key parameters in eqn (2) are likely to vary among taxonomic groups and environmental gradients via acclimation and/or evolution (Padfield *et al*. 2016, 2017; Scafaro *et al*. 2017; Schaum *et al*. 2017), our approach treats MST as a model framework that captures important processes that determine how individual‐level traits shape emergent community‐level properties such as GPP, CR and the individual size distribution. Using the ML approach, we tested whether the metabolic normalisation constant, $b\left(T_{c}\right)$, activation energy, *E*, and individual size‐scaling exponent, *α*, differed between communities exposed to long‐term warming by comparing the goodness‐of‐fit of models with and without a term for long‐term warming (warm or ambient mesocosm) for each estimated parameter. Model selection was carried out by comparing nested models using likelihood ratio tests. Analyses were carried out in R using the package ‘*bbmle’* (Bolker & Team 2010), with separate models for GPP and CR.

We then assessed how well the MST framework could approximate our measurements of community metabolism. To do this, we used eqn (1) and the best fit parameter estimates ($b\left(T_{c}\right)$, *E* and *α*) to calculate temperature‐corrected metabolic rate and mass‐corrected biomass for GPP and CR of each community. According to eqn (1), we expect temperature‐corrected community metabolic rate to be directly proportional to mass‐corrected community biomass (i.e. have a slope of 1 when both variables are log‐transformed). To test this, we used standardised major axis (SMA) regression to quantify the slope of the relationship between temperature‐corrected metabolic rate and mass‐corrected biomass (Warton *et al*. 2006). SMA was useful here as we were not interested in predicting one variable from another, but rather estimating the underlying line of best fit between the variables (we are testing for an expected slope of 1) (Warton *et al*. 2006).

We used the individual size distribution of each community to investigate whether shifts in abundance, biomass and size structure of communities compensated for the direct effects of temperature on individual metabolic rate, buffering the response of total community metabolism to warming. We estimated average individual‐level gross photosynthesis using the parameters estimated from the ML fits for GPP and then dividing the estimate of metabolism (calculated using eqn (2)) by total community abundance ($\bar{m_{i}^{\alpha }}=\frac{M_{\mathrm{tot}}\left(m_{i}^{\alpha -1}\right)e^{E(\frac{1}{kT_{c}}-\frac{1}{\mathrm{kT}})}}{n_{\mathrm{tot}}}$). Thus, $\bar{m_{i}^{\alpha }}$ accounts for the effect of temperature, changes in the metabolic normalisation constant and size structure on average individual‐level metabolic rate. We tested the slope of the log‐log relationship between average individual metabolic rate and abundance using SMA regression against the expected slope −1. For all SMA regression analyses, confidence intervals of the predictions were created by bootstrapping 1000 replicates of the data and model.

## Results

### Effect of warming on community composition and size structure

**Figure 1:**
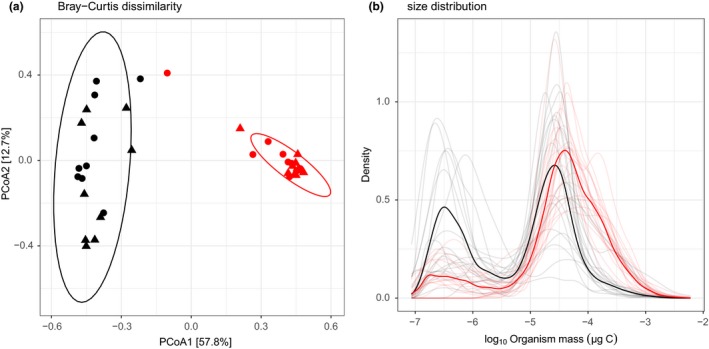
Effects of long‐term warming on community structure. (a) Principal Coordinate (PCoA) plot of communities based on Bray–Curtis distance. The percentage of variation explained is shown on each axis (calculated from the relevant eigenvalues). Long‐term warming alters community composition while short‐term warming had no impact. (b) Probability density function for the size distribution of ambient and warm mesocosms. Warm mesocosms are dominated by larger phytoplankton. In both panels, different colours are used to represent ambient (black) and warm (red) mesocosms. In (a) triangles represent the warm incubator and circles, the ambient incubator; ellipses represent the 95% confidence interval ellipses of the ambient and warm mesocosms. In (b) the pronounced line represents the size distribution of the pooled communities, while the faded lines show the size distribution of each individual community.

Long‐term warming significantly altered the composition of the phytoplankton communities (i.e. the Bray–Curtis dissimilarity among communities, Fig. 1a, permanova,* F*_1,36_ = 41.62, partial *R* ^2^ = 0.54, *P* = 0.0001). In contrast, short‐term warming had no effect on community composition (permanova,* F*_1,36_ = 1.66, partial *R* ^2^ = 0.02, *P* = 0.154). The difference in composition altered community size structure with communities from the ambient mesocosms having fewer large phytoplankton (mean size = 3.6 × 10^−5^ μg C) compared to those from the warm mesocosms (mean size = 1.06 × 10^−4^ μg C) (Fig. 1b).

### Effect of short‐ and long‐term warming on community metabolism

**Figure 2:**
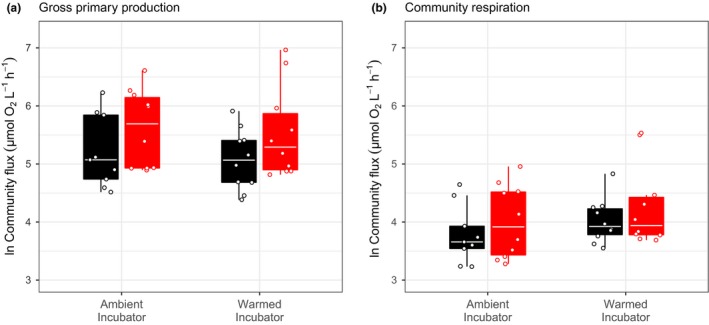
The effect of short‐ and long‐term warming on (a) gross primary production and (b) community respiration. The maximum likelihood framework found that short‐term warming increases GPP and CR in both ambient (black) and warm (red) mesocosms. Long‐term warming resulted in large shifts in community composition and size structure. However, there was no significant effect of either short‐ or long‐term warming on either GPP or CR. Each point represents the metabolic flux of one community; tops and bottoms of box‐whisker plots represent the 75th and 25th percentiles and the white horizontal line represents the median.

Total gross primary production and community respiration did not significantly change as a result of either short‐ or long‐term warming (Fig. 2, Table S1). This is consistent with ‘community‐level metabolic compensation’, where adjustments to abundance and size structure buffer the direct effects of temperature on total metabolic rates.

### Temperature dependence and size‐scaling of community metabolism

We fitted eqn (2) to the measurements GPP and CR (Fig. 2 & Table 1). After accounting for differences in size structure between communities, we quantified the effects of short‐term warming on community metabolic rate and found that the temperature dependence of gross primary production (*E*_*GPP*_ = 0.74 eV [*Q*_10_ = 2.75]; 95% CI = 0.20–1.29 eV) was weaker than that of community respiration (*E*_*CR*_ = 1.42 eV [*Q*_10_ = 6.98]; 95% CI = 0.85–1.98 eV). Using the maximum likelihood approach, we also estimated the size‐scaling exponent of gross primary production (*α*_*GPP*_ = 0.87; 95% CI = 0.58–1.17) and community respiration (*α*_*CR*_ = 1.14; 95% CI = 0.74–1.41) which were not significantly different (they have overlapping 95% confidence intervals) from previously found three quarters, or isometric (*α *= 1) scaling that has been found in phytoplankton. Long‐term warming had no impact on the temperature dependence, size‐scaling and metabolic normalisation constant of GPP or CR (Table 1 & Table S2).

**Table 1:** Results of the maximum likelihood model fitting, which entailed estimating the parameters below simultaneously by fitting eqn (2) to gross primary production, community respiration and the community size distribution

Parameter	Units	Estimate	95% confidence interval
*E*_*GPP*_	eV	0.61	0.11–1.12
*E*_*CR*_	eV	1.27	0.70–1.83
*α*_*GPP*_	–	0.88	0.57–1.17
*α*_*CR*_	–	0.80	0.31–1.18
$\ln GPP\left(T_{c}\right)$	μmol O_2_ L^−1^ h^−1^	−3.46	−6.27 to −1.02
$\ln CR\left(T_{c}\right)$	μmol O_2_ L^−1^ h^−1^	−5.50	−10.10 to −2.22

### Estimating gross primary production and community respiration from metabolic scaling theory

**Figure 3:**
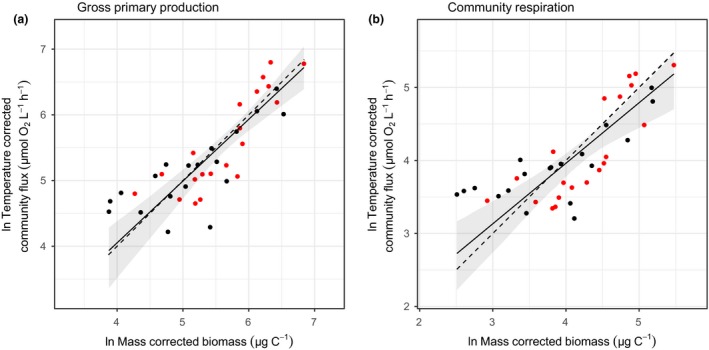
Relationship between (a) temperature‐corrected community gross primary production and mass‐corrected community biomass, and (b) temperature‐corrected community respiration and mass‐corrected community biomass. Community metabolism can be predicted from changes in community abundance, body size and short‐term temperature. Rates were temperature normalised using the empirically derived values of E_GPP_ = 0.61 eV in (a) and E_CR_ = 1.27 eV in (b). Mass‐corrected biomass was calculated using the values of α and $b\left(T_{c}\right)$ from the maximum likelihood approach (Table 1). The warm mesocosm communities are denoted by red points and the ambient mesocosms by black points. The solid lines and shaded regions represent the predictions and 95% confidence intervals of standardised major axis regression investigating whether the slope between observed and expected metabolism is significantly different from the predicted slope of 1. The dashed line represents a 1 : 1 line as predicted from metabolic scaling theory.

After estimating the temperature dependence and size‐scaling of metabolic rate for GPP and CR, we used eqn (1) to calculate mass‐corrected community biomass, $M_{\mathrm{tot}}\left(m_{i}^{\alpha -1}\right)$, and the temperature‐corrected community rates, $B_{j}\left(T\right)e^{E\left(\frac{1}{kT_{c}}-\frac{1}{\mathrm{kT}}\right)},$ of GPP and CR for each microcosm using the parameter estimates from the best fit maximum likelihood model (Table 1). After accounting for the effect of short‐term warming and mass on metabolic rate, our scaling approach predicted that temperature‐corrected rates should increase proportionally (1 : 1) with mass‐corrected biomass on a log‐log scale (Fig. 3; dashed lines). For GPP, the SMA regression had an intercept of 0.29 (95% CI = −0.68–1.26), a slope of 0.94 (95% CI = 0.78–1.13) and an *R* ^2^ of 0.68 (Fig. 3a). For CR, the SMA regression had an intercept of 0.64 (95% CI = −0.09–1.37), a slope of 0.83 (95% CI = 0.67–1.02) and an *R* ^2^ of 0.59 (Fig. 3b). The relationships between mass‐corrected biomass and temperature‐corrected rate for both GPP and CR had values of the slopes and intercepts that were not significantly different from the expected values (slope = 1; intercept = 0). This suggests that metabolic scaling theory provides a reasonable representation of total community metabolism from information on the size distribution and the environmental temperature (Fig. 3). Fitting the same model using OLS regression returned similar results.

### Community‐level metabolic compensation

We calculated the average individual metabolic rate for each community using the parameter values for GPP from the maximum likelihood approach (Table 1; see Methods) and quantified the relationship between total community abundance and average individual metabolic rate using SMA regression. Total community abundance was inversely correlated with average individual metabolic rate with a slope (slope = −1.16, 95% CI = −1.59 to −0.85; intercept = 3.47, 95% CI = −1.03 – 7.96; *R* ^2^ = 0.21; Fig. 3) that was not significantly different from the predicted value of −1 (test comparing slope to ‐1 by testing the correlation between residual and fitted values, *r* = 0.16, d.f. = 37, *P* = 0.35). This result is consistent with our expectations based on community‐level metabolic compensation – that is, a trade‐off between the number of organisms and the average individual‐level metabolic rate – and demonstrates that shifts in abundance, biomass and size structure compensate for the direct effects of high temperature on metabolic rates.

## Discussion

Current methods of measuring planktonic metabolism *in situ* and *in vitro* have led to disagreement as to whether aquatic systems are net autotrophic or heterotrophic (del Giorgio & Peters 1994; del Giorgio *et al*. 1997; Duarte *et al*. 2011; Williams *et al*. 2013). These differences are driven, in part, by the different mechanisms that influence metabolism at the contrasting scales at which the measurements are taken. Previous work has attempted to address these differences using metabolic scaling theory to predict phytoplankton community metabolism by linking individual physiology and community size structure (del Giorgio *et al*. 1999; López‐Urrutia *et al*. 2006; Yvon‐Durocher & Allen 2012). Here, we extend this work by examining both the direct and indirect effects of warming on phytoplankton community metabolism by empirically testing a model that links phytoplankton GPP and CR with the effects of body size and temperature on individual physiology and community size structure. Long‐term warming resulted in marked differences in community composition, which shifted communities towards larger phytoplankton species, altering the size structure between communities (Fig. 1). This effect is thought to be driven by stronger top‐down control in the warm mesocosms through elevated zooplankton grazing (Yvon‐Durocher *et al*. 2015). Despite differences in composition and size structure between communities, rates of both GPP and CR were well predicted from the individual size distribution and the effects of body size and temperature on individual metabolic rate (Fig. 3).

In line with previous work, gross primary production was less sensitive to temperature change than community respiration (Table 1), with the activation energies of GPP and CR at the community level similar to those recently found for single species of phytoplankton at the population level (i.e. gross photosynthesis and respiration) (Padfield *et al*. 2016; Schaum *et al*. 2017). Previous studies have used the short‐term temperature sensitivities of GPP and CR to predict the impact of long‐term warming on phytoplankton communities (López‐Urrutia *et al*. 2006). As respiration increases relative to photosynthesis in the short‐term, the metabolic balance of phytoplankton communities are thought to shift towards heterotrophy (López‐Urrutia *et al*. 2006; Regaudie‐De‐Gioux & Duarte 2012; Yvon‐Durocher & Allen 2012), which could potentially exacerbate further climate warming. However, using short‐term responses to temperature to predict the impacts of long‐term warming (as is expected of climate warming) on community metabolism is not straightforward. In the long‐term, warming can indirectly influence community metabolic flux through changes in phytoplankton composition, size structure (Yvon‐Durocher *et al*. 2011; Dossena *et al*. 2012) standing biomass (Yvon‐Durocher *et al*. 2015) and the individual‐level metabolic normalisation constant (Padfield *et al*. 2017; Scafaro *et al*. 2017).

**Figure 4:**
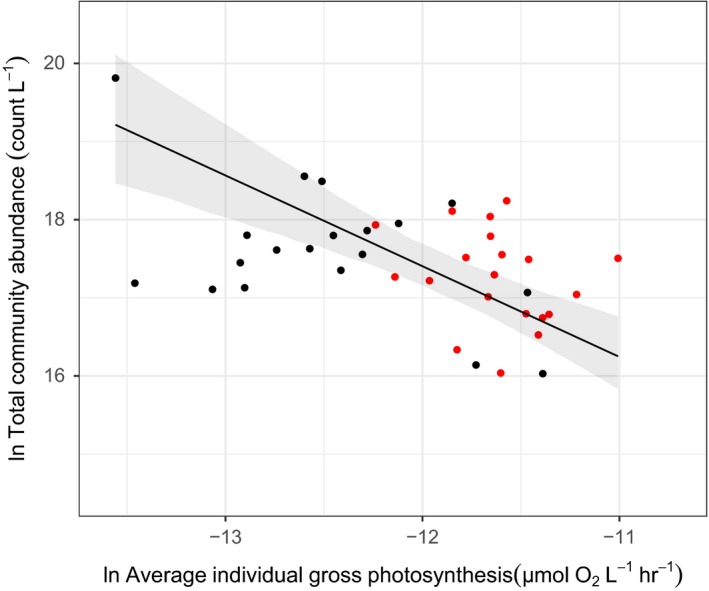
Total community abundance decreases as average individual gross photosynthesis increases. Average individual gross photosynthesis was calculated from the values of the maximum likelihood approach for gross primary productivity (see Methods). Consistent with zero sum dynamics, increases in average individual gross photosynthesis resulted in a compensatory decrease in total community abundance. The exponent, fitted using standardised major axis regression on log‐transformed data, is −1.16, which is not significantly different from the predicted value of −1. Warm mesocosms (red) have higher average individual metabolic rates than ambient mesocosm communities (black) because of being dominated by larger individuals. Each point represents a community, the solid line is the predicted fit of the SMA regression and the shaded region are 95% confidence intervals.

In our experiment, warming and changes in size structure did not affect total community metabolism. This somewhat paradoxical result was due to a trade‐off between community properties, where differences in temperature and shifts in size structure between communities were compensated for by changes in total community abundance. At steady state, MST predicts a trade‐off between total abundance and average individual metabolic rate. Such ‘community‐level metabolic compensation’ means that communities at different temperatures with different composition and size structure can have similar metabolic rates because an ultimate energetic constraint (e.g. the carrying capacity of the microcosm) sets an upper limit on community metabolism (White *et al*. 2004; Ernest *et al*. 2008, 2009). In line with this prediction, across all the communities, microcosms with higher average individual rates of gross photosynthesis had proportionally lower community abundance (Fig. 4). Community‐level metabolic compensation meant that higher average individual metabolic rates, due to larger phytoplankton or higher temperatures, were compensated for by changes in total abundance, resulting in no overall change in community metabolism (Fig. 2). This indirect effect of warming on community abundance adds to recent work highlighting the importance of considering ecological and evolutionary processes that can indirectly alter metabolic rates when predicting the response of ecosystems to warming (Michaletz *et al*. 2014; Yvon‐Durocher *et al*. 2015; Padfield *et al*. 2017; Schaum *et al*. 2017).

Overall, this method, derived from metabolic scaling theory, successfully inferred rates of GPP and CR from the individual size distribution and environmental temperature (Fig. 3). This is because community metabolism is ultimately driven by the abundance, temperature and individual size distribution of the organisms that comprise the community. The success of this simple theoretical framework in explaining variation in community metabolism suggests that aggregate ecosystem functions can be understood from information on the individual size distribution and the temperature dependence of metabolic rate, irrespective of the taxonomic identities of the organisms that comprise the communities. Our approach is not truly predictive in that it requires estimation of the key parameters characterising the size and temperature dependence of metabolic rate. However, this is not necessarily a weakness as recent evidence suggests that these parameters are not universal constants, but rather vary among taxonomic groups and different environments owing to physiological acclimation and evolutionary adaptation (Padfield *et al*. 2016; Reich *et al*. 2016; Scafaro *et al*. 2017; Schaum *et al*. 2017; Vasseur *et al*. 2012). Further progress in understanding taxonomic and environmental variation in the size and temperature dependence of metabolic rate and developing theory for predicting this variation from first principles will be vital to improving the scope and application of metabolic scaling theory in ecological research.

## Author contributions

G. Y‐D. and D.P. conceived the study and designed the experimental work. D. P. and R.W. conducted the experiments and D.P. and C.L. did the DNA extractions and the bioinformatics. D.P. analysed the data and D.P., A.B., C.L. and G.Y.D. contributed to writing the paper. The authors declare no conflict of interest.

## Data accessibility statement

All data and R code used for the analysis is available on GitHub https://git.io/vpzfu. This repository includes the raw sequence data from the experiment.
