# Supplementary material for: Linking phytoplankton community metabolism to the individual size distribution
Source: Ecol Lett. 2018 May 25;21(8):1152–61. doi: 10.1111/ele.13082 (PMC6849760; doi:10.1111/ele.13082)
Supplement: Supplementary file 1 [file ELE-21-1152-s001.pdf]

Supplementary Information:

Linking phytoplankton community metabolism to the individual size distribution

Daniel Padfield[1], Angus Buckling[2], Ruth Warfield[1], Chris Lowe[2], & Gabriel Yvon-Durocher[1]

Author affiliations

[1] Environment and Sustainability Institute, University of Exeter, Penryn, Cornwall, TR10 9EZ, U.K.

[2] Centre for Ecology and Conservation, College of Life and Environmental Sciences, University of Exeter, Penryn, Cornwall, TR10 9FE, U.K.

Corresponding author: Daniel Padfield (d.padfield@exeter.ac.uk)

Author contributions: G. Y-D. and D.P. conceived the study. D.P. and G. Y-D. designed the experimental work. D. P. and R.W. conducted the experiments and D.P. and C.L. did the DNA extractions and the bioinformatics analyses. D.P. analysed the data and D.P., A.B., C.L. and G.Y.D. contributed to writing the paper. The authors declare no conflict of interest.

Data accessibility statement: All data and R code used for the analysis will be made available on GitHub.

Section 1. Supplementary Tables and Figures.

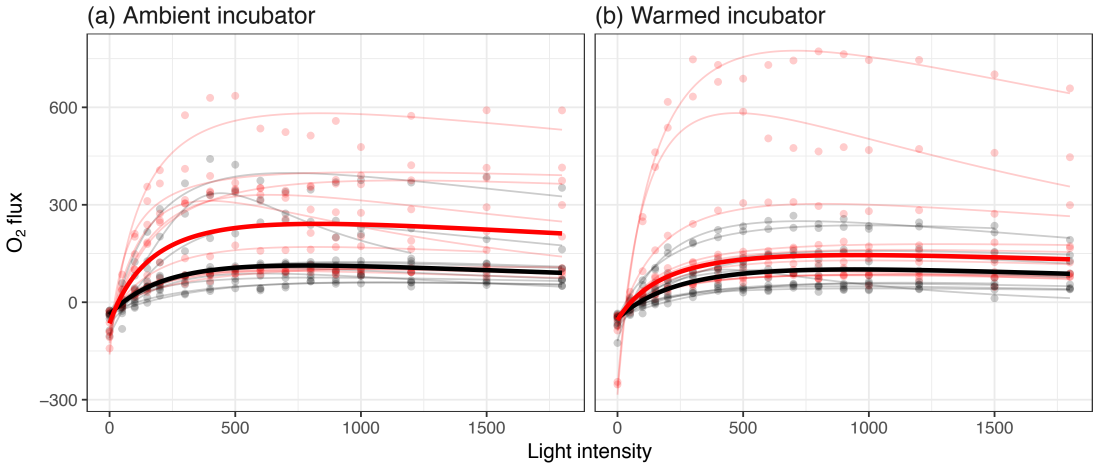

Figure S1. Photosynthesis irradiance curves of warm (red) and ambient (black) mesocosm communities in the (a) ambient and (b) warmed incubators. Values for the photosynthetic maximum (Eq S1) were used as the metabolic rate data to which Eq 2 was fitted. Faded points and lines represent the raw measurements and individual fits to each photosynthesis irradiance curve. The bold, thicker lines represent the fit of the average parameter values from all of the individual parameter values.

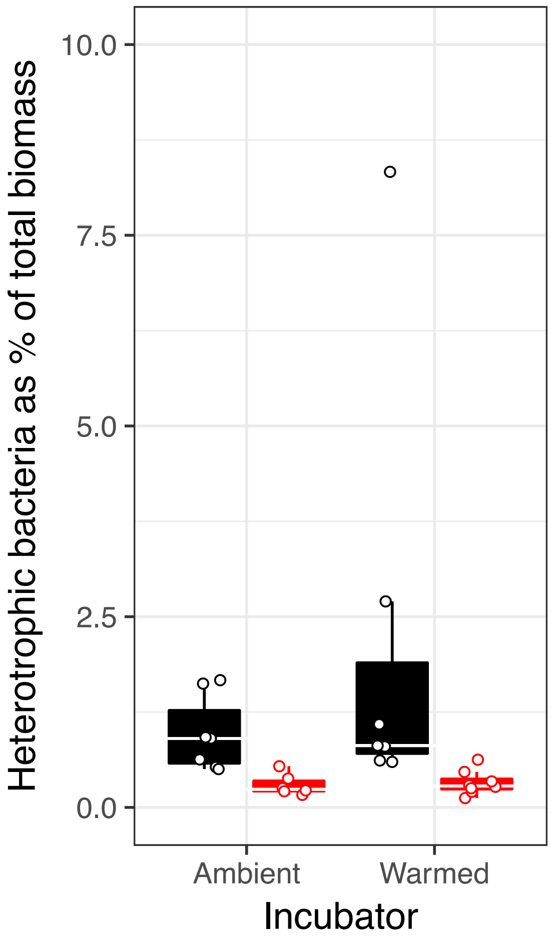

Figure S2. Proportion of heterotrophic bacteria of total biomass. The proportion of heterotrophic bacteria of total biomass is less than 3% in both warm and ambient mesocosms in all but a single community. Consequently, all analyses only used the size distributions from the autotrophic communities. Each point represents one community; tops and bottoms of box-whisker plots represent the 75th and 25th percentiles and the white horizontal line represents the median.

Table S1. Results of mixed effects model analysis for the effects of short- and long-term warming on community metabolic rate. Analyse reveal that community flux is not significantly altered by either short- or long-term warming. The most parsimonious model after model selection is highlighted in bold.

flux	model	d.f.	AIC	Log Lik	χ^2	P
gross primary production	Random effects structure					
	~ 1|mesocosm					
	Fixed effects structure					
	1. ~ 1 + short-term warming * long-term warming	6	84.59	-36.29		
	2. ~ 1 + short-term warming + long-term warming	5	82.61	-36.30	0.021	0.88
	3. ~ 1 + long-term warming	4	80.95	-36.47	0.34	0.55
	**4. ~ 1**	3	82.661	-38.33	3.71	0.054
community respiration	Random effects structure					
	~ 1|mesocosm					
	Fixed effects structure					
	1. ~ 1 + short-term warming * long-term warming	6	71.35	-29.67		
	2. ~ 1 + short-term warming + long-term warming	5	69.35	-29.68	0.0071	0.93
	3. ~ 1 + long-term warming	4	68.73	-30.37	1.38	0.24
	**4. ~ 1**	3	69.71	-31.86	2.98	0.08

Table S2. Results of model selection for the maximum likelihood model for simultaneously estimating parameters in Eq. 2. The most parsimonious model after model selection is highlighted in bold.

flux	model	d.f	AIC	Deviance	χ^2	P
gross primary production	1. long-term warming * E_{GPP} + long-term warming * α_{GPP} + long-term warming * $GPP(T_c)$	6	56.45	44.49		
	2. E_{GPP}+ long-term warming * α_{GPP} + long-term warming * $GPP(T_c)$	5	55.9	45.91	1.46	0.23
	3. E_{GPP} + α_{GPP} + long-term warming * $GPP(T_c)$	4	52.96	44.96	0.94	0.33
	**4. $\boldsymbol{E_{GPP} + \alpha_{GPP} + GPP(T_c)}$**	3	51.3	45.3	0.34	0.56
community respiration	1. long-term warming * E_{CR} + long-term warming * α_{CR} + long-term warming * $CR(T_c)$	6	60.86	48.86		
	2. E_{CR} + long-term warming * α_{CR} + long-term warming * $CR(T_c)$	5	58.92	48.92	0.07	0.8
	3. E_{CR} + α_{CR} + long-term warming * $CR(T_c)$	4	57.62	49.63	0.7	0.4
	**4. $\boldsymbol{E_{CR} + \alpha_{CR} + CR(T_c)}$**	3	59.31	53.31	3.67	0.054

Section 2. Supplementary Methods

Additional methods on mesocosm setup, measurements of metabolic flux and the individual size distribution

Overview of long-term mesocosm experiments

Twenty freshwater mesocosms, each holding 1 m^3, were set up in 2005 to mimic shallow lake ecosystems. They are situated at the Freshwater Biological Association's river laboratory (2º 10' W, 50º 13' N) in East Stoke, Dorset, UK. Of the twenty, ten mesocosms have been warmed by 4 ºC above ambient temperature for more than 10 years. The mesocosms were seeded in December 2005 with organic substrates and a suite of organisms from surrounding natural freshwater habitats and subsequently left open to natural colonisation. These mesocosms have previously shown that warming can alter community structure and the metabolic balance of ecosystems (Dossena *et al.* 2012; Yvon-Durocher *et al.* 2015), and continue to provide a powerful tool to investigate how individual- and community-level properties influence ecosystem functioning and the impact of warming on these links.

Experimental setup and maintenance

We sampled all twenty mesocosms (~200 mL) and inoculated each sample into laboratory microcosms in a reciprocal transplant experiment on 13th April 2016. Microcosms were inoculated with a starting density of 200 cells mL^{-1} in water collected from the mesocosms supplemented with Bold's Basal Medium (BBM) (0.01 of standard concentration) and placed in incubators (Infors-HT) at 16 ºC and 20 ºC (the temperatures of the ambient and warmed mesocosms respectively on the day of sample collection). Each mesocosm was passed through a 40 µm filter prior to inoculation to remove zooplankton from the microcosms. Phytoplankton communities were maintained on a 12:12 light:dark cycle with a daily light intensity of 175 μmol^{-1} m^{-2} s^{-}

[1]. This resulted in 40 communities with 10 replicates of each combination of short- and long-term warming (i.e. warmed mesocosm in warm incubator, ambient mesocosm in ambient incubator, warmed mesocosm in ambient incubator and ambient mesocosm in warm incubator). Autotroph counts were tracked daily using flow cytometry (BD Accuri C6). After ~17 days of culture, most communities showed a slowing of biomass accrual due to density dependence and resource limitation. The communities were then maintained by replacement of 50% of the culture with new medium (mesocosm water supplemented with BBM).

Analysing sequencing data

Sequence data was analysed in R (v 3.3.2) (Team 2014) using the packages *'dada2'* and '*phyloseq'* (Callahan *et al.* 2015, 2016). Reads were truncated at 250 bp. We then followed the full stack workflow to estimate error rates, infer and the merge sequences, construct a sequence table, remove chimeric sequences and assign taxonomy (Callahan *et al.* 2016). Sequence inference was done by pooling all the samples to improve the detection of rare variants that are seen just once or twice in an individual sample, but many times across all samples. We combined multiple rRNA databases to create a new database from which taxonomy was assigned. PhytoREF (Decelle *et al.* 2015), provides a reference database for the plastidial 16S rRNA gene for photosynthetic eukaryotes. Consequently, in a single amplicon sequencing run of the 16S v4 region we quantified both bacterial and eukaryotic autotroph diversity. We combined the PhytoREF database with the Ribosomal Database Project (Cole *et al.* 2014) that contains ribosomal RNA sequences of prokaryotes and 2700 16S rDNA cyanobacterial references (Decelle *et al.* 2015). Using CD-HIT (Li & Godzik 2006) we created a clustered database that aligned sequences with >97% similarity. We then preferentially assigned clustered sequences

as originating from 1) PhytoREF, 2) cyanobacteria or 3) the Ribosomal Database Project as previous work has shown erroneous assignments of chloroplast plastidial sequences as being of bacterial origin (Decelle *et al.* 2015). We used the R package '*taxise'* (Chamberlain & Szöcs 2013) to reconcile each species in the reference database with its higher taxonomy. Samples were removed if represented by fewer than 1000 reads and the remaining samples were standardised to the total number of amplicon sequence variants (ASVs; Callahan *et al.* 2017) through rarefaction to account for biases associated with differences in sequencing effort. We then selected ASVs (amplicon sequence variants) corresponding to autotrophic taxa, which resulted in samples from 37 of the 40 communities that could be used for downstream analysis.

Measuring community metabolism

After ~30 days of culture (enough time for acclimation responses to short-term warming to occur [1-10 generations in phytoplankton] (Staehr and Birkeland, 2006)), we measured metabolism at incubator temperature (16 ºC or 20 ºC) on communities below carrying capacity. Aliquots (30 mL) of each community were concentrated through centrifugation (~1500 rpm for 30 minutes at 4 ºC) and resuspended in 5 mL and acclimatised to the measurement temperature for 15 minutes in the dark prior to measuring metabolic flux. Primary production and community respiration were measured through oxygen evolution in the light and oxygen consumption in the dark respectively on a Clark-type electrode (Hansatech Ltd, King's Lynn UK Chlorolab2). Primary production was measured at increasing light intensities in minutely intervals of 50 μmol^{-1} m^{-2} s^{-1} to 200 μmol^{-1} m^{-2} s^{-1} and then in intervals of 100 μmol^{-1} m^{-2} s^{-1} up to 1800 μmol^{-1} m^{-2} s^{-1} that yielded a photosynthesis irradiance (PI) curve (Figure S1). Rates of community respiration were measured for two minutes in the dark at the end

of each PI curve to ensure respiration was not limited by available photosynthate during the measurement period.

Each individual PI curve was fit to a modification of the Eiler's curve for photoinhibition that incorporates community respiration. This model allows for negative rates of net primary production at low light levels even when community respiration is greater than gross primary production (Eilers and Peeters, 1988):

$$NPP(I) = \frac{NPP_{max} I}{(NPP_{max}/\alpha I_{opt}{}^{2})\, I^{2} + \left(1 - \left(\frac{2NPP_{max}}{\alpha I_{opt}}\right)\right) I + \frac{NPP_{max}}{\alpha}} - CR \quad \text{(S1)}$$

where $NPP(I)$, is the rate of net primary production at irradiance, I, NPP_{max} is the maximal rate of net primary production at optimal light, I_{opt}, α controls the gradient of the initial slope and CR is community respiration, the rate of oxygen consumption in the dark. Gross primary production (GPP) at light saturation was then found by adding community respiration onto maximal net primary production ($GPP = NPP_{max} + CR$). Gross primary production at light saturation and measured community respiration were subsequently used in the metabolic scaling framework.

References

Callahan, B.J., Mcmurdie, P.J., Rosen, M.J., Han, A.W., Johnson, A.J. & Holmes, S.P. (2015). DADA2 : High resolution sample inference from amplicon data. *bioRxiv*, 13, 0–14.

Callahan, B.J., Sankaran, K., Fukuyama, J.A., McMurdie, P.J. & Holmes, S.P. (2016). Bioconductor workflow for microbiome data analysis: from raw reads to community analyses. *F1000Research*, 5, 1492.

Chamberlain, S.A. & Szöcs, E. (2013). taxize: taxonomic search and retrieval in R. *F1000Research*, 2.

Cole, J.R., Wang, Q., Fish, J.A., Chai, B., McGarrell, D.M., Sun, Y., *et al.* (2014). Ribosomal Database Project: Data and tools for high throughput rRNA analysis. *Nucleic Acids Res.*, 42, 633–642.

Decelle, J., Romac, S., Stern, R.F. & Bendif, E.L.M. (2015). PhytoREF : a reference database of the plastidial 16S rRNA gene of photosynthetic eukaryotes with curated taxonomy, 1435–1445.

Dossena, M., Yvon-Durocher, G., Grey, J., Montoya, J.M., Perkins, D.M., Trimmer, M., *et al.* (2012). Warming alters community size structure and ecosystem functioning. *Proc. R. Soc. B Biol. Sci.*, 279, 3011–3019.

Li, W. & Godzik, A. (2006). Cd-hit: a fast program for clustering and comparing large sets of protein or nucleotide sequences. *Bioinformatics*.

Team, R.C. (2014). R: A language and environment for statistical computing. R Foundation for Statistical Computing, Vienna, Austria, 2012.

Yvon-Durocher, G., Allen, A.P., Cellamare, M., Dossena, M., Gaston, K.J., Leitao, M., *et al.* (2015). Five years of experimental warming increases the biodiversity and productivity of phytoplankton. *PLoS Biol.*, 13, 1–22.
